# Systematic reviews on behavioural and psychological symptoms in the older or demented population

**DOI:** 10.1186/alzrt131

**Published:** 2012-07-11

**Authors:** Rianne M van der Linde, Blossom CM Stephan, George M Savva, Tom Dening, Carol Brayne

**Affiliations:** 1 Department of Public Health and Primary Care - Forvie Site, Institute of Public Health, University of Cambridge, Robinson Way, Cambridge CB2 0SR, UK; 2 The Irish Longitudinal Study on Ageing, Trinity College Dublin, Dublin 2, Republic of Ireland; 3 Cambridgeshire and Peterborough NHS Foundation Trust, Box 311, Fulbourn Hospital, Cambridge CB21 5EF, UK

## Abstract

**Introduction:**

Behavioural and psychological symptoms of dementia (BPS) include depressive symptoms, anxiety, apathy, sleep problems, irritability, psychosis, wandering, elation and agitation, and are common in the non-demented and demented population.

**Methods:**

We have undertaken a systematic review of reviews to give a broad overview of the prevalence, course, biological and psychosocial associations, care and outcomes of BPS in the older or demented population, and highlight limitations and gaps in existing research. Embase and Medline were searched for systematic reviews using search terms for BPS, dementia and ageing.

**Results:**

Thirty-six reviews were identified. Most investigated the prevalence or course of symptoms, while few reviewed the effects of BPS on outcomes and care. BPS were found to occur in non-demented, cognitively impaired and demented people, but reported estimates vary widely. Biological factors associated with BPS in dementia include genetic factors, homocysteine levels and vascular changes. Psychosocial factors increase risk of BPS; however, across studies and between symptoms findings are inconsistent. BPS have been associated with burden of care, caregiver's general health and caregiver depression scores, but findings are limited regarding institutionalisation, quality of life and disease outcome.

**Conclusions:**

Limitations of reviews include a lack of high quality reviews, particularly of BPS other than depression. Limitations of original studies include heterogeneity in study design particularly related to measurement of BPS, level of cognitive impairment, population characteristics and participant recruitment. It is our recommendation that more high quality reviews, including all BPS, and longitudinal studies with larger sample sizes that use frequently cited instruments to measure BPS are undertaken. A better understanding of the risk factors and course of BPS will inform prevention, treatment and management and possibly improve quality of life for the patients and their carers.

## Introduction

Behavioural and psychological symptoms (BPS) include depressive symptoms, anxiety, apathy, sleep problems, irritability, psychosis, wandering, elation and agitation. They are common in people with dementia, but are not restricted to this group [1]. BPS have public policy implications as they impact upon quality of life of older people and their carers, and influence prescribing and use of services [2,3].

Systematic reviews are an important tool for summarising the available evidence regarding a specific topic, permitting policy decisions to be based on representative literature. They allow researchers to focus on areas where information is most lacking, and allow more reliable interpretation of research findings. A review of reviews, therefore, serves two important purposes: first, summarising the evidence base on a subject beyond the scope of a single review, and second, highlighting areas where the literature is inadequate and where additional reviews are required. While the prevalence, course, biological and psychosocial associations, care and outcomes of BPS have been the subject of systematic reviews, the findings from these different reviews have not been brought together in this way. Given the broad research focus on BPS, including underlying causes, association with dementia risk, and impact on care, and the large number of studies published in this area, any single systematic review of the primary literature can only explore a narrow range of objectives.

This paper presents a systematic 'review of reviews' of the literature on BPS in older people. Combining current knowledge across the multiple domains of BPS research provides a broad overview of what is known and identifies gaps where reviews have not been conducted [4]. Bringing together the conclusions of the reviews, we provide recommendations for future research and highlight areas where the evidence base with respect to BPS in the older population should be strengthened. In addition, we discuss the recommendations for future research made by the reviews.

## Materials and methods

### Scope of review

BPS are related to cognitive impairment and dementia. So-called "behavioural and psychological symptoms of dementia", BPSD, are commonly studied within this subpopulation, but BPS can also occur in older people without significant cognitive impairment. Traditionally these are considered as phenomena distinct to BPSD; however, BPS in a cognitively healthy older person may indicate early dementia, and certain BPS, for example, depression, may be risk factors for dementia. Continuities in BPS are seen 'pre' and 'post' diagnosis, and common biological and psychosocial risk factors for BPS may exist among the cognitively healthy and cognitively impaired older populations. For this reason the scope of our review includes studies of BPS in the older population with or without cognitive impairment or dementia. We included reviews of the prevalence, the causes and consequences of BPS.

**Figure 1:**
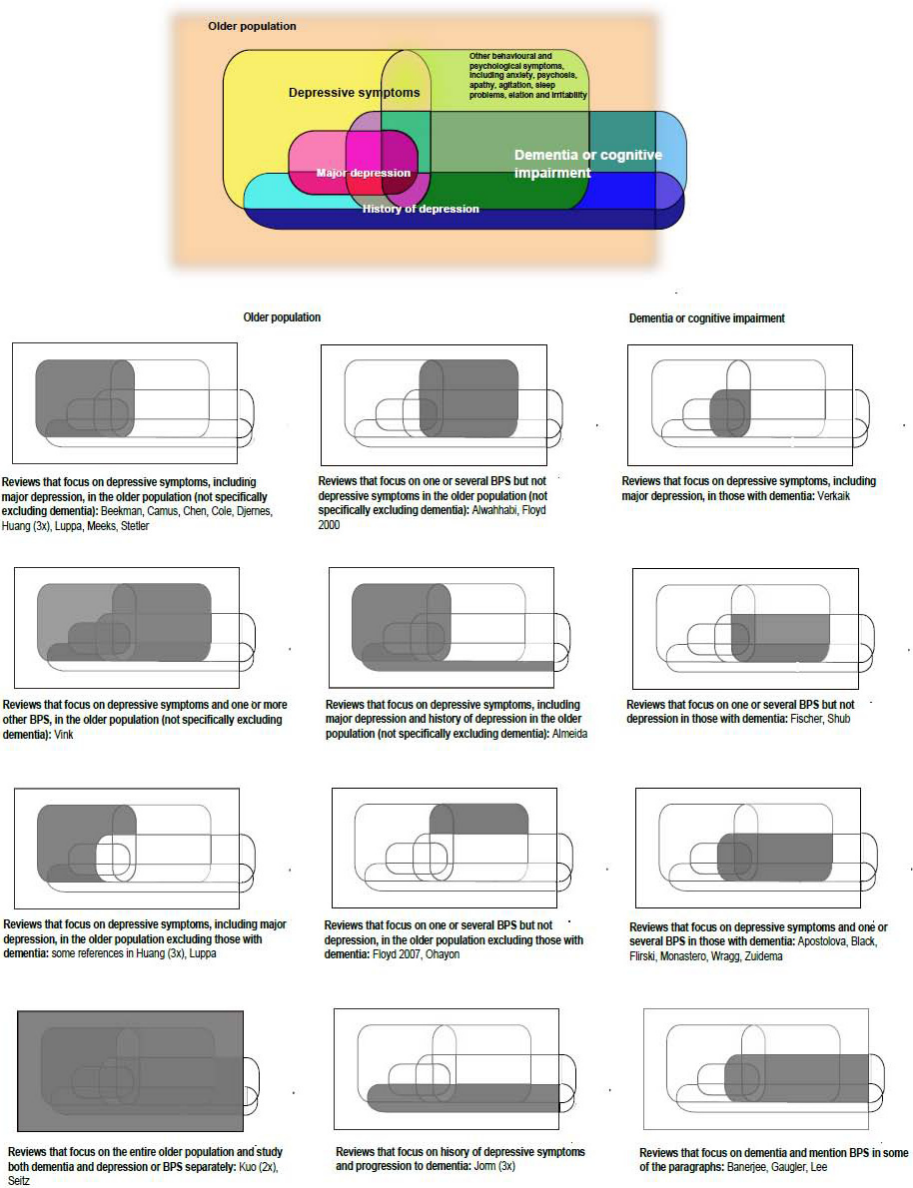
**Populations and BPS that were the focus of the reviews**.

Owing to the breadth of literature and the specialist treatment required to review certain kinds of study, there are some limitations to the scope of this review. We did not include reviews that focused on pharmacological or non-pharmacological treatment of symptoms. Depression is a heterogeneous disorder ranging from mild symptoms to major depressive disorder. Depression is common in the older population with dementia and can be studied in the context of other BPS, but is also seen in the older population without dementia (Figure 1). Depression in the older population without dementia has been studied widely since it was first described in 1896 [5,6]. The term BPSD was introduced by the International Psychogeriatric Association in 1996 [7]. Although they have been identified since the earliest descriptions of dementia, research only moved to BPSD in the 1980s with the development of instruments to measure BPSD [8,9]. Depression as a BPSD and depression in the older population without dementia have largely been separate research areas. Here, we focused on depressive symptoms below the threshold for depressive disorder. We excluded reviews that studied only major or clinical depression and included reviews that studied both major depression and minor depression, depressive symptoms or minor depression only or depressive symptoms in the context of other BPS.

### Search methods

Embase and Medline were searched for potentially relevant articles published before 29 March 2012. Search terms included Emtree terms and text searches for each individual BPS and BPS in general (see Additional file 1), and Dementia (Emtree) or Aged (Emtree). Additional articles were identified from reference lists of included studies and relevant narrative reviews.

### Data collection

All systematic reviews written in English of one or more BPS in the older non-demented or demented population were included. A systematic review was defined as used by the Cochrane Collaboration and the Preferred Reporting Items for Systematic Reviews and Meta-Analyses (PRISMA) Statement: "A review of a clearly formulated question that uses systematic and explicit methods to identify, select, and critically appraise relevant research, and to collect and analyse data from the studies that are included in the review. Statistical methods (meta-analysis) may or may not be used to analyse and summarise the results of the included studies" [10]. Specific symptoms included depressive symptoms (including sadness, tearfulness, being unhappy, depressed feeling, suicidal feelings), anxiety (including feelings and physical signs of anxiety, worrying, being frightened), apathy (including listlessness, loss of interest, slowing), sleep problems (includes reduced sleep, increased sleep, change in sleep, tiredness), irritability (including being irritable or angry, verbal and physical aggression), psychosis (including delusion and hallucination), wandering (including wandering away, getting lost, aimless wandering), elation (including euphoria, inappropriate laughing) and non-aggressive agitation (including restlessness, repetitive behaviour).

RvdL selected articles through a multi-step screening process first based upon the assessment of the title and the abstract, followed by assessment of the article content. Information from each potential article was extracted by RvdL using a standardised form, recording: the BPS investigated, population, date of publication and literature search, number of studies reviewed and if a meta-analysis was performed. Reviews were divided by the following themes: prevalence, progression, course, biological associations, risk factors, care, quality of life and disease outcome. Results were summarised using the abstract. A list of recommendations for future research (for example, "future research should", "we recommend", "is needed") and limitations of the original studies and review as reported by the reviews in their discussion section was generated.

The quality of the included reviews was assessed using AMSTAR, a validated measurement tool (Shea et al. 2007, Ottawa, Ontario, Canada) to assess the methodological quality of systematic reviews [11,12].

## Results

### Number of studies

Separate searches of each BPS resulted in 266 reviews for depressive symptoms (*N *= 156 in dementia), 110 for anxiety (*N *= 74 in dementia), 8 for apathy (*N *= 7 in dementia), 82 for sleep problems (*N *= 45 in dementia), 30 for irritability (*N *= 24 in dementia), 42 for psychosis (*N *= 33 in dementia), 3 for wandering (*N *= 3 in dementia), 3 for elation (*N *= 3 in dementia), 15 for agitation (*N *= 14 in dementia) and 29 for BPS (*N *= 28 in dementia). Altogether, 399 reviews were found. Of these, 28 reviews were included. The others were excluded because BPS were not the main focus of the paper, instead they studied treatment or non-pharmacological interventions, did not focus on elderly or dementia populations, were not performed systematically or did not meet any other inclusion criteria. Seven reviews were excluded because they only studied major depression [13-19]. Reference searches of the included systematic reviews and relevant narrative reviews identified nine additional reviews. Therefore, in total 36 reviews were included.

### Characteristics and focus of included reviews

Tables 1 and 2 show the characteristics of the reviews included. Most (*N *= 13) [20-32] investigated the prevalence or co-occurrence of symptoms. Eleven [21,27,30,33-40] reviewed the longitudinal course of BPS or its associations with incident dementia. Possible underlying biological factors were examined in nine reviews [41-49], as were psychosocial risk factors [20,24,26,27,30,31,49,50]. Only two reviews [51,52] focused on the associations between BPS and care outcomes, one [53] on the effects on quality of life and four [30,32,54,55] on disease outcomes.

**Table 1:** Characteristics of included reviews and summary of findings - older population with dementia or cognitive impairment

First author	Search date	BPS	Popu-lation	N reviewed	Summary of results	Meta-analysis	Recommendations future research	Reported limitations	Quality
**Prevalence and co-occurrence**									

Monastero [21]	Aug 2008	BPS	MCI	27	Prevalence: 35 to 85%. Most common: dep, anx and irr. Hospital-based studies reported higher prevalence than population based studies	-	- Large cohort studies - Using standardised MCI criteria - Standardised behavioural instruments - Prognostic role of BPS in MCI - Not exclude those with dep at baseline Previously proposed: - Prevalence - Correlates - Different MCI subtypes, - Genetic/biological markers	*Original studies* - Differences in methodology, including setting, age and sex distribution, inclusion and exclusion criteria and differential sensitivity of BPS instruments *Review* - English language only - Heterogeneity affected ability to compare data	4

Apostolova [22]	Dec 2006	BPS	MCI	21	Prevalence: 35 to 75%. Most common BPS: dep, apa, anx and irr. Least common: ela, hal, dis and wan	-	- Large, prospective longitudinal studies - Standard MCI diagnostic criteria not excluding those with depression - Neuropsychiatric instruments designed for the cognitively impaired	*Original studies* - Different sampling methods - Exclusion of subjects with depression - Heterogeneity of MCI diagnostic criteria - Heterogeneity of BPS instruments *Review* - Only English literature - Heterogeneity limits comparison	3

Seitz [23] See 1B	Mar 2009	BPS Dep, Anx	Care home	35	Prevalence BPS in dementia: 78% (median)	-	See Table 2	See Table 2	3

Zuidema [24]	Aug 2005	BPS	MMSE < 24, care home	25	Prevalences ranged considerably, from 3 to 54% for del, 1 to 39% for hal, 8 to 74% for dep, 7 to 69% for anx, 17 to 84% for apa, 48 to 82% for agg or agi, and 11 to 44% for psychical agg.	-	See risk factors	See risk factors	2

Shub [25]	NR	Psy Agg	Dem	54	Of seven cross and two long studies directly examining correlation agg and psy, most showed a positive association.	-	- Prospectively designed studies - Temporal relationship	*Original studies* - Limited data of sufficient methodological rigor - Majority cross-sectional studies	3

Wragg [26]	NR	Dep Psy	Dem	30	Dep and Psy occurred in 30 to 40% of AD patients. Isolated symptoms were two to three times as frequent as diagnosable affective or psychotic disorders. Paranoid del were the most common psy symptoms.	-	- Specify an a priory hypothesis - Clearly report characteristics study sample - Prospective and longitudinal design - Use standardised case definitions - Reliable and valid BPS instruments - Use appropriate statistical analysis - Clearly report conclusions - Both theoretical and clinical focus.	*Original studies* - Methodological limitations including small samples, misclassification bias *Review* - Relatively small number of studies spanning several decades - Publication bias	2

Ropacki [27]	2003	Psy	Dem	55	Prevalence psy: 41% (del 36%, hal: 18%)	-	- Longitudinal designs - Incidence and persistence of psychosis - Develop or utilise diagnostic criteria and rating scales for psychosis - Take into account medication use - Mechanisms underlying psychosis in AD	*Original studies* - Severely cognitively impaired subjects not included - Potential effects of medication on cognition - Small standard deviations age at onset and illness duration - Assessing psy: diagnostic criteria nonspecific to dementia and inconsistencies interpreting criteria	1

**Course and progression**									

Monastero [21]	Aug 2008	BPS	MCI	27	Prospective studies showed that BPSD, particularly depression, may represent risk factors for MCI or predictors for the conversion of MCI to AD.	-	See prevalence	See prevalence	4

Verkaik [34]	Mar 2006	Dep	Dem	24	1/4 (continous) and 0/3 (categorical) high quality studies found a significant association between severity of AD and prevalence of dep.	-	- Longitudinal study - Using a standardised dementia definitions - Assessment severity of AD - Assessing dep with dem specific instrument - Control for confounders	*Review* - Only English language, studies that did not have depression or depressive disorder as a keyword were not identified	5

Ropacki [27]	2003	Psy	Dem	55	Incidence increased progressively the first three years, after that plateau. Duration several months but less prominent after one year. Associated with more rapid cognitive decline	-	See prevalence	See prevalence	1

**Biological**									

Flirski [42]	?	BPS	Dem	73	Behavioural genetics of BPS reviewed: genes coding for APOE E, serotonin receptors, serotonin transporter, COMT, MAO-A, tryptophan hydroxylase and dopamine receptors. A general conclusion is the striking inconsistency of the findings, unsurprising in the field of psychiatric genetics.	-	- Precisely define symptoms - Fusing multidisciplinary data	*Original studies* - Inconsistency of results - Recruitment solely based on clinical diagnosis - Variability in study design - BPS fluctuation, studies rely heavily on average disease state - Cross sectional studies - Variety of BPS instruments - Studying isolated symptoms or symptom clusters - Selection bias: ethnicity or genetic homogeneity, choice of setting - Insufficient number of study participants - No correction for multiple testing - Carrier status versus dose - Multifactorial aetiology	0

**Risk factors**									

Zuidema [24]	Aug 2005	BPS	MMSE < 24, care home	25	BPSD predicted not only by dem type or stage, but also by the psychosocial environment and the amount of psychoactive medication and physical restraints used.	-	- Effects of manipulation the physical and social environments in nursing homes.	*Original studies* - Uncertainty defining dem, its type and severity - Few accurately diagnosed dementia - Different BPS instruments and definitions	2

Wragg [26]	NR	Dep Psy	Dem	30		-	See prevalence	See prevalence	2

Ropacki [27]	2003	Psy	Dem	55	Associations: age, age at onset AD, illness duration. Weak/inconsistent: gender, education, family history dem or psychiatric illness	-	See prevalence	See prevalence	1

**Care**									

Gaugler [51]	2006	BPS	Dem	80	Behavioural symptoms one of most consistent predictors of nursing home admission in persons with dementia.	-	- Interventions should consider long-term efficacy and timing of nursing home admission in course of dem - Power for subgroup analyses - More complex models of institutionalisation.	*Review* - Research synthesis method: requires descriptive information from samples composed of subjects a similar age; studies providing only correlations and not means or standard deviations are excluded and some assumption made about shape of distribution.	5

Black [52]	Dec 2001	BPS	Dem	55	Pooled correlation coefficients for relationship BPS and caregiver burden (0.57, 95%CI 0.52 to 0.62), caregiver psychological stress (0.41, 0.32 to 0.49) and caregiver depression (0.30, 0.21 to 0.39). Multivariate data supported BPS are predictor of burden of care, psychological distress and dep. Limited long data. Caregiver variables may be more important in predicting institutionalisation than BPS.	See summary of results	- Concept of burden of care is too broad and more clinically relevant measures such as caregiver depression are preferred. - Cohort studies	*Original studies* - Relatively few studies - Majority clinic-based samples, few representative - Majority cross-sectional and correlational - Little about which care-recipient symptoms are most distressing or particular risk factors for subgroups - Concept of burden may be too broad - Other variables are likely to be important *Review* - Publication bias - Pooling data assumes homogeneity (questionable)	3

**Disease outcome**									

Lee [54]	NR	BPS	Dem	NR	There was no consensus regarding the association with dementia prognosis	-	- Guideline for dementia prognostication - Risk score to better estimate survival.	*Original studies* - Uncertainty of etiologic diagnosis of dementia	1

Fischer [55]	NR	Psy	Dem	6	Three of six studies showed and association with real-world functioning	-	- Longitudinal studies - More detail about delusional severity, - Use cognitive and functional measures that are better at detecting executive impairment to clarify the association.	*Original studies* - Basic measures functional performance + cognition - Confounders not always taken into account - Considerable variation definition del - Psychoactive medication not taken into account - No longitudinal studies	2

**Quality of life**									

Banerjee [53]	Oct 2007	BPS	Dem	NR	Strong suggestion dep is consistently associated with decreased health related quality of life in dem. Magnitude of associations is moderate and the proportion of variance explained is low.	-	- Quality of life in dementia: determinants, in dementia subtypes, self- versus proxy-report, in different settings, association with outcomes and interventions	NR	0

AD, Alzheimer's disease; Agg, aggression; Anx, anxiety; Biol, biological associations; BPS, behavioural and psychological symptoms; Cross, Cross-sectional; Dem, Dementia; Dep, depressive symptoms; Ela, elation; Long, longitudinal; MCI, mild cognitive impairment; Psy, psychosis; Sle, sleep problems

**Table 2:** Characteristics of included reviews and summary of findings - general older population

First author	Search date	BPS	Popu-lation	N reviewed	Summary of results	Meta-analysis	Recommendations future research	Reported limitations	Quality
**Prevalence and co-occurrence**									

Seitz [23] See 1A	Mar 2009	BPS Dep Anx	Care home	35	Prevalence dep symptoms in long term care: 29% (14 to 82%)	-	- Developing countries - Multinational studies - Collaboration across centres - Adoption of standard survey methods - Effective and safe interventions	*Original studies* - Small sample size - Conducted in developed countries - Included relatively few long term care facilities - Many studies conducted several years ago	3

Luppa [28]	May 2010	Dep	Older (60+)	24	Prevalence of dep disorders ranged from 4.5 to 37.4%. Pooled prevalence: 17.1% (95% CI 9.7 to 26.1)	Pooled prev major dep: 7.2% (95%CI 4.4 to 10.6) Dep disorders: 17.1 (9.7 to 26.1)	- Large scale - Population-based - Prospective studies - Also covering oldest age segments - Comorbidity, cognition and function - Suitable depression diagnostics	*Original studies* - Methodological differences in study design, sampling structure and study quality	5

Chen [20]	Jun 1997	Dep	Older (60+)	10	Prevalence dep mood: 14.8 (14.2 to 15.6%), higher in rural communities	Prev dep mood: 14.8% (14.2 to 15.6)	- Similar methodology - Culture-specific validated instruments - Risk factors and understanding dep	*Original studies* - Much variation - Cultural acceptability of instruments	4

Beekman [29]	1996	Dep	Older, community dwelling (55+)	34	The reported prevalence rates vary enormously (0.4 to 35%). Minor dep: 9.8% (8.3 to 14.3) Clinical dep symptoms: 13.5% (2.8 to 35%)	-	- Focus on those most at risk and in adverse socio-economic conditions - Improving comparability of the data	*Original studies* - Methodological differences - Bias translating instruments *Review* - Formal meta-analysis was not considered justified	3

Meeks [30]	Jan 2010	Dep	Older (55+)	153	Dep was generally at least two to three times more prevalent than major dep. Prevalence lower in community settings (9.8%, 4.0 to 22.9) than primary care (15.1 to 35.9%) and LTC (4.0 to 30.5%).	-	- Incidence - Prevalence - Various clinical settings, - Diverse geographical areas - Cultural/socioeconomic groups - Neurobiology - Treatment - Terminology of depression - Associations with psychopathology	*Review* - Could not conduct a meta-analysis due to data heterogeneity - Review did not include data on early or mild adulthood subthreshold depression, limiting extrapolation of findings to other age groups	2

Djernes [31]	Sep 2004	Dep	Older (65+)	122	Prevalence clinical relevant depressive symptoms: 7.2 to 49%	-	- Target risk factors, improvement of prevention and treatment of chronic somatic and mental illnesses, adequate social support, prevention social isolation - Education and information dep in elderly - Comparability of methodology - Focus on nursing home residence	*Original studies* - Methodological differences - Rates of participation; depressed elderly may be particularly prone to refuse research invitations - Subjective variations in the assessment of the presence or absence of a diagnostic criterion - Differences between instruments	2

Alwahhabi [32]	2001	Anx	Older (55+)	119		-	See disease outcome	See disease outcome	1

**Course and progression**									

Huang [35]	Aug 2007	Dep	Older (55+)	17	Non-dementia cognitive impairment vs without: incidence dep: OR = 1.5, 95% CI 0.9 to 2.5 prevalence dep: RR = 1.1, 95% CI 0.6 to 2.0. Dem vs. no dem: incidence OR = 1.8, 85% CI 1.2 to 2.9, prevalence RR = 3.9, 95% CI 1.9 to 8.0	See summary of results	- Risk for cognitive impairment for depression	*Review* - No conclusion if dep was risk factor for dem - No hand-search of journals and no attempt to identify unpublished studies. English language only - Heterogeneity among included studies - Confounding comorbidity other psychiatric disorders - Data only gathered until august 2007 - Only four longitudinal studies included	5

Meeks [30]	Jan 2010	Dep	Older (55+)	153	8 to 10% of subthreshold dep developed major dep per year. Median remission rate to non-dep status 27% after > 1 year.	-	- Longitudinal course	See prevalence	2

Jorm [33,36,37]	End 2000	Dep	Dem/Older	11, 15, 2	1991: history of dep (late onset cases) associated with AD (late onset). 2000: Dep increased risk of dem in case control, 95% CI 1.2 to 3.5 and prospective studies, 95% CI 1.1 to 3.2.; 2001: Update 2000: case control studies: RR = 2.0, 95% CI 1.2 to 3.5, prospective studies 1.9, 95% CI 1.1 to 3.2	Too many results	1991: - Prospective studies - History of psychiatric disorders other than dep and psychiatric treatments 2000/2001: - Large sample size - Mechanisms association dep and dem	*Review* 1991 - The pooled analyses cover only a small number of exposures from the domain of psychiatric history	0

Ohayon [38]	2003	Sle	Adult ("healthy or normal")	65	Total sleep time, sleep efficiency, percentage of slow-wave sleep, percentage of REM sleep and REM latency all significantly decreased with age. Sleep latency, waking after sleep, waking after sleep duration and the percentage of stage 1 and 2 sleep increase with age, but only sleep efficiency continued to significantly decrease after 60 yr.	Age - sleep: TST: r = -0.76 *P *< 0.0001 Sleep efficiency: r = -0.82, %SWS: r = -0.56% REM: r = 0.16 Sleep latency: r = 0.16% stage 1 sleep: r = 0.16% stage 2 sleep: r = 0.34 WASO: r = 0.75 All *P *< 0.0001	- Strict screening methods - Effect of race - Take into account subjects' habitual sleep schedules as well as whether PSG recording occurs on weekday or weekend night	*Original studies* - No information given in relation with the presence or absence of sex differences, no information about race composition - Several studies did not include middle-aged subjects *Review* - Limited to peer-reviewed studies	3

Floyd [39]	2002	Sle	Adult ("healthy or normal")	244	Age and REM%: essentially linear, decreasing 0.6% per decade but ceased during mid-70s followed by small increase 75 to 85	Age - REM%: r = -0.17	- REM sleep in women - More data in old-old population	*Review* - Studies did not screen for psychoactive substance use, dep and sleep apnea, few studies of women - Univariate approach - Publication bias	2

Floyd [40]	1996	Sle	Adult	41	Night-time sleep amount and the ability to initiate sleep decreased with age. Larger age-related changes when sleep variables were measured by polysomnography rather than self-report.	Age - sleep, effect size: Sleep latency: 0.19 (0.14 to 0.24) WASO frequency: 0.38 (0.34 to 0.42) WASO duration: 0.74 (0.71 to -0.77) Night time sleep amount: -0.33 (-0.37 to -0.28)	- Controlling for health moderators (carefully assessed for levels of depression, sleep apnea and use of psychoactive substances) - Study women	*Original studies* - Inclusion or exclusion of certain covariates may have influenced which predictors emerged as significant - Very few of the studies examined the effects of collinearity, moderation or mediation among critical predictor variables - Range of quality scores *Review* - Heterogeneity made the estimation of pooled effects impractical	1

**Biological**									

Huang [43]	Aug 2007	Dep	Older (55+)	28	Significant OR and RR for increased dep in old age: stroke, loss of hearing, loss of vision, cardiac disease or chronic lung disease had a. Significant OR but un-significant RR: arthritis, hypertension and diabetes. Both OR and RR not significant: gastro-intestinal disease	Too many results		*Review* - Not hand-search journals, not identify unpublished studies, three databases, only English language - Risk factors dep might be differently related to the onset, chronicity and recurrence but not differentiated - Recent life event not taken into account - Heterogeneity in results	5

Huang [44]	Aug 2007	Dep	Older (55+)	31	Chronic disease - dep: RR = 1.5, 95% CI 1.2 to 2.0. poor SRH - dep: RR = 2.4, 95% CI 1.9 to 3.0.	Chronic disease - dep: RR = 1.5 (1.2 to 2.0) SRH - dep: RR = 2.4 (1.9 to 3.0)		*Review* - Not hand-search journals, no attempt to identify unpublished studies, three databases, only English - Heterogeneity in results	5

Almeida [45]		Dep	Older (70+)	17	High tHcy increased risk of dep: OR = 1.7, 95% CI 1.4 to 2..1 TT vs. CC carriers: OR = 1.2, 95% CI 1.0 to 1.5	High tHcy - dep: OR = 1.7 (1.4 to 2.1) MTHFR C677T - dep: TT vs CC: OR = 1.2 (1.0 to 1.5) CT vs CC: OR = 1.1 (0.9 to 1.2)	- Sufficiently powered randomised trials	*Original studies* - Small sample size (trials) - Reverse causality (observation studies) - Inconsistent definition phenotype, misclassification bias (genetic studies) - Lack of reliable information on ethnicity *Review* - Meta-analysis lacked power	4

Stetler [46]	May 2009	Dep	Adult	414	Dep vs no dep: Cortisol d = 0.6 (95% CI 0.5 to 0.7) Adrenocorticotropic-releasing hormone d = 0.28 (95% CI 0.2 to 0.4) Corticotropin-releasing hormone d = 0.02 (95% CI -0.5 to 0.5)	Too many results	- Bioinformatic technologies - Larger sample size - Longitudinal	*Original studies* - High degree of heterogeneity - Publication bias possible - Based on cross-sectional studies - Arbitrary criteria for minimal methodological quality - Most of the included studies were underpowered	3

Kuo [47]	Sep 2004	Dep	Adult	19	High concentrations C-reactive protein predictive of cognitive decline and dem. Relations to dep cross and not consistent.	-	- Prospective study c-reactive protein-dep - Intervention studies to lower c-reactive protein and improved outcomes	NR	3

Kuo [41]	Mar 2004	Dep	Older (55+)	NR	Growing evidence of association hyper-homocysteinemia and cognitive impairment, dem and dep. Proposed mechanisms include angiotoxicity, neurotoxicity, and inhibition of collagen cross-linking	-	- Role of homocysteine in prevention - Prospective studies association with dep - Adequate adjustment for possible confounders	NR	3

Camus [48]	Jun 2003	Dep	Older	NR	Potential ways association dep - vascular disease: 1 direct influence vascular disease, 2 direct influence dep, 3 common causes	-	- Pathophysiological and genetic background of vascular depression	NR	1

Vink [49]	Dec 2005	Anx Dep	Older (50+)	80	Risk factors anx and dep showed many similarities but some differences were found. Biological factors may be more important in predicting dep, and a differential effect of social factors on dep and anx was found.	-	- Intervention (whether manipulation of risk factors reduces the onset of anx/dep) - Clearer understanding of etiological factors differentiating anx and dep	*Review* - Heterogeneity between studies, no meta-analysis - Only main effects of risk factors on anx and dep - Heterogeneity limits comparison across studies - Risk factors that have not yet been studied - No distinction made between different anx disorders	1

**Risk factors**									

Chen [20]	Jun 1997	Dep	Older (60+)	10	The patterns of risk factors were similar to those in western countries	See prevalence	See prevalence	See prevalence	4

Meeks [30]	Jan 2010	Dep	Older (55+)	153	Risk factors: female, medical burden, disability and low social support; neurological illnesses (Parkinson's disease, stroke, AD)	-	- While some risk factors are well established, others remain to be identified.	See prevalence	2

Djernes [31]	Sep 2004	Dep	Older (65+)	122	Risk factors: female, somatic illness, cognitive and functional impairment, lack of social contacts, history of dep	-	See prevalence	See prevalence	2

Cole [50]	2001	Dep	Older (50+)	20	Risk factors, Qualitative: disability, new medical illness, poor health status, prior depression, poor self-perceived health, and bereavement. Quantitative: bereavement, sleep disturbance, disability, prior depression, female gender	13 risk factors investigated. OR ranged from 1.0 to 3.3, significant risk factors: bereavement, sleep disturbance, disability, prior dep, female gender	- Intervention	*Original studies* - Follow-up incomplete in most studies - Differences in the length of follow-up - Differences in definitions risk factors and adjustment - Many potential risk factors not studied adequately - Cumulative effect of multiple risk factors not studied - Heterogeneity in the results *Review* - Search by one author only - Only English or French literature - Did not assess publication bias - Abstracted by one author	2

Vink [49]	Dec 2005	Anx, Dep	Older (50+)	80	Risk factors both anx and dep: personality, coping strategies, previous psychopathology, social network, stressful life events, female. Dep: smaller network size, being unmarried.	-	See biological	See biological	1

**Disease outcome**									

Meeks [30]	Jan 2010	Dep	Older (55+)	153	Consequences: disability, greater healthcare utilisation, increase suicide ideation	-	- More sophisticated health economic studies	See prevalence	2

Alwahhabi [32]	2001	Anx	Older (55+)	119	Limitations: understanding expression anx, variable definitions elderly, diagnostic instruments. Anx in elderly potential for negative consequences independent of comorbidity major dep.	-	- Definition of elderly - Symptom definition and diagnostic instruments - Clinical trials	*Original studies* - No common definition of the lower limit of geriatric age	1

AD, Alzheimer's disease; Agg, aggression; Anx, anxiety; BPS, behavioural and psychological symptoms; Cross, Cross-sectional; Dem, Dementia; Dep, depressive symptoms; Ela, elation; Long, longitudinal; MCI, mild cognitive impairment; Prev, prevalence; Psy, psychosis; Sle, sleep problems; SRH, self-rated health

Figure 1 shows the populations and BPS that were the focus of the different reviews. As shown, most reviews focused on depression in the older population, not specifically excluding those with dementia (*N *= 11) [20,29-31,35,43,44,46,48,50,56], or depression and several other BPS in dementia (*N *= 6) [21,22,24,26,42,52]. Of the studies focusing on depressive symptoms or other BPS in the older population, it was often unclear if those with dementia were included, with only few specifically including only studies of "healthy" or "normal" elderly [38,39] or reporting which original studies excluded those with dementia or cognitive impairment [28,35,43,57]. Overall, 12 reviews [20,28-32,41,45,48-50] included studies of the entire older population (> 50 years), 3 [35,43,44] of the older population but with some studies excluding those with dementia or cognitive impairment, 1 [23] of patients in long-term care, 3 [40,46,47] of an adult population including older aged adults, 2 [38,39] that only included 'healthy' adults, and 3 included case control studies comparing those with dementia to normal controls [33,36,37]. In addition, 3 [21,22,24] studied cognitively impaired populations and 10 [25-27,34,42,51-55] people with dementia. Nine reviews included a wide battery of BPS [21-24,42,51-54] and a further three [25,26,49] focused on a combination of two or more symptoms. Of those studying a single symptom, depressive symptoms were most widely reviewed (*N *= 18) [20,28-31,33-37,41,43-48,50]. Sleep problems (*N *= 3) [38-40], psychosis (*N *= 2) [27,55] and anxiety (*N *= 1) [32] were the subject of few reviews. Details of the characteristics and recruitment of the population of the original studies included in the reviews were often not reported.

### Quality

The quality assessment for each included review is shown in Table 3. Generally, review quality was low, with only 7 of the reviews scoring positive on 5 of the 11 components and meeting the criteria for moderate scientific quality (5 to 8 points). All other reviews scored less than five points.

**Table 3:** Methodological quality of systematic reviews assessed with the AMSTAR measurement tool

Author	1 A priori design	2 Duplicate	3 Search	4 Publication status	5 List of studies	6 Characteristics studies	7 Scientific quality reported	8 Conclusions	9 Combination methods	10 Publication bias	11 Conflict of interest	Score	Quality
Almeida [45]	Can't answer	Can't answer	No	No	No	Yes	Yes	No	Yes	Yes	No	4	Low
Alwahhabi [32]	Can't answer	Can't answer	No	No	No	No	Yes	No	Not applicable	No	No	1	Low
Apostolova [22]	Can't answer	Can't answer	No	No	No	Yes	Yes	Yes	Not applicable	No	No	3	Low
Banerjee [53]	Can't answer	Can't answer	Can't answer	No	No	No	No	No	Not applicable	No	No	0	Low
Beekman [29]	Can't answer	Can't answer	No	Can't answer	No	Yes	Yes	Yes	Not applicable	No	No	3	Low
Black [52]	Can't answer	Can't answer	Can't answer	No	No	Yes	Yes	Yes	No	No	No	3	Low
Camus [48]	No	Yes	No	No	No	No	No	No	Not applicable	No	No	1	Low
Chen [20]	No	Can't answer	No	Yes	No I	Yes	No	Yes	Yes	No	No	4	Low
Cole [50]	Can't answer	No	Can't answer	No	No	Yes	Yes	No	No	No	No	2	Low
Djernes [31]	Can't answer	Can't answer	Can't answer	Can't answer	No	Yes	Yes	No	Not applicable	No	No	2	Low
Fischer [55]	Can't answer	Can't answer	Can't answer	No	No	Yes	No	Yes	Not applicable	No	No	2	Low
Flirski [42]	Can't answer	Can't answer	No	Can't answer	No	No	No	No	Not applicable	No	No	0	Low
Floyd [40]	Can't answer	Can't answer	Can't answer	No	No	No	No	No	Yes	No	No	1	Low
Floyd [39]	Can't answer	Yes	Can't answer	Yes	No	No	No	No	No	No	No	2	Low
Gaugler [51]	Can't answer	Yes	Can't answer	Yes	No	Yes	Yes	Yes	Not applicable	No	No	5	Moderate
Huang [35]	Can't answer	Yes	Can't answer	Can't answer	No	Yes	Yes	No	Yes	Yes	No	5	Moderate
Huang [43]	Can't answer	Yes	Can't answer	Can't answer	No	Yes	Yes	No	Yes	Yes	No	5	Moderate
Huang [44]	Can't answer	Yes	Can't answer	No	No	Yes	Yes	No	Yes	Yes	No	5	Moderate
Jorm [33,36,37]	No	Can't answer	Can't answer	Can't answer	No	No	No	No	No	No	No	0	Low
Kuo [47]	Can't answer	Yes	No	No	No	Yes	No	Yes	Not applicable	No	No	3	Low
Kuo [41]	Can't answer	Can't answer	No	No	No I	Yes	No	Yes	Not applicable	No	No	3	Low
Lee [54]	Can't answer	Can't answer	No	No	No	Yes	No	No	Not applicable	No	No	1	Low
Luppa [28]	Can't answer	No	Can't answer	No	No	Yes	Yes	Yes	Yes	Yes	No	5	Moderate
Meeks [30]	Can't answer	Can't answer	No	No	No	Yes	No	Yes	Not applicable	No	No	2	Low
Monastero [21]	Can't answer	Yes	No	No	No	Yes	Yes	Yes	Not applicable	No	No	4	Low
Ohayon [38]	Can't answer	Can't answer	Can't answer	No	No	Yes	Yes	Yes	No	No	No	3	Low
Ropacki [27]	Can't answer	Can't answer	Can't answer	No	No	Yes	No	No	Not applicable	No	No	1	Low
Seitz [23]	Can't answer	Yes	Can't answer	Can't answer	No	Yes	No	Yes	Not applicable	No	No	3	Low
Shub [25]	Can't answer	Can't answer	No	No	No	Yes	Yes	Yes	Not applicable	No	No	3	Moderate
Stetler [46]	Can't answer	No	No	No	No	Yes	No	Yes	No	No	No	3	Low
Verkaik [34]	Yes	Yes	Can't answer	No	No	Yes	Yes	Yes	Not applicable	No	No	5	Moderate
Vink [49]	Can't answer	Can't answer	Can't answer	No	No	Yes	No	No	Not applicable	No	No	1	Low
Wragg [26]	Can't answer	Can't answer	No	No	No	Yes	No	Yes	Not applicable	No	No	2	Low
Zuidema [24]	Can't answer	Can't answer	No	Can't answer	No	Yes	No	No	Not applicable	No	No	2	Low

Response options: yes, no, can't answer, not applicable.

Full questions: *A priori *design: Was an "a priori" design provided? Duplicate: Was there duplicate study selection and data extraction? Search: Was a comprehensive literature search performed? Publication status: Was the status of publication (that is, grey literature) used as an inclusion criterion? List of studies: Was a list of studies (included and excluded) provided? Characteristics studies: Were the characteristics of the included studies provided? Scientific quality reported: Was the scientific quality of the included studies assessed and documented? Conclusion: Was the scientific quality of the included studies used appropriately in formulating conclusions? Combination methods: Were the methods used to combine the findings of studies appropriate? Publication bias: Was the likelihood of publication bias assessed? Conflict of interest: Was the conflict of interest stated? Score: The maximum AMSTAR score a review can receive is 11 (11 for meta-analyses and 10 for systematic reviews) Quality: Scores of 0 to 4 indicated low quality, 5 to 8 moderate quality, and 9 to 11 high quality.

### Narrative description by theme

#### Prevalence of BPS

Two reviews [21,22] of BPS in populations with mild cognitive impairment (MCI) reported an overall symptom prevalence ranging from 35 to 75% and 35 to 85%. Depressive symptoms, anxiety and irritability were the most commonly observed symptoms, followed by apathy and agitation. Hospital-based samples of MCI cases reported a higher mean prevalence of any BPS than population-based studies [21]. In people with dementia in long term care the prevalence of one or more BPS was 78% [23]. The mean reported prevalence of psychosis in dementia was 41% [27].

Reviews of the prevalence of BPS in the older population without dementia were available only for depression. The prevalence of depressive disorders in the older population without dementia has been estimated to be 17.1% (95% CI 9.7 to 26.1), in a meta-analysis of moderate scientific quality [28]. Other studies reported a high variability in the prevalence reported in included studies ranging from 14 to 82% [23], 14 to 16% [20], 0 to 35% [58] and 7 to 49% [31]. A lower prevalence has been reported in community settings than in primary care and long term care settings [30].

Most reviews found considerable variability in reported prevalence, possibly due to heterogeneity in methodology, thereby limiting the ability to compare data. Recommendations include the use of standardised BPS instruments and the study of various populations.

#### Longitudinal course and association with cognitive decline

Monastero *et al. *reported that prospective studies showed that BPS, particularly depression, might represent risk factors for MCI or predictors of MCI conversion to dementia [21]. A review of moderate scientific quality failed to find evidence of an association between depressive symptoms and severity of dementia [34]. A review on psychotic symptoms suggested that these symptoms increase with the development of dementia but plateau after three years [59].

Few reviews on BPS course have been conducted in the older population. Huang *et al. *reported that compared to individuals without cognitive impairment, incidence and prevalence of depression was higher in those with cognitive impairment or dementia. Meeks *et al. *found that depression was relatively stable in the older population, with a median remission of 27% after more than one year [30]. Three reviews by Jorm *et al. *concluded that history of depression in cognitively normal persons was associated with increased risk of dementia [33,36,37,60]. Sleep problems including sleep latency and waking after sleep were common with increasing age, but only sleep efficiency continued to significantly decrease after age 60 [38-40].

The reviews identified a need for more longitudinal studies using standardised measures of cognitive function and BPS and appropriate adjustment for confounding factors.

#### Biopsychosocial associations

##### Biological factors

A systematic review shows inconsistent results for genetic associations, including genes coding for APOE E, serotonin receptors and transporter, COMT, MAO-A, tryptophan hydroxylase and dopamine receptors with BPS in individuals with dementia [42]. No other reviews were found.

In the general older population, most reviews of biological correlates of BPS focus on depression. An association between high levels of homocysteine and depression and dementia has been reported [41,45]. Stetler *et al. *suggest an association between depression and cortisol and other hormones [46]. In addition, cerebral atherosclerotic changes may result in cognitive impairment and depression, possibly mediated by C-reactive protein but results were not consistent [47]. The association between vascular factors and depression was further studied and discussed in a review by Camus *et al. *[48].

Recommendations for future research include prospective studies with large sample sizes further investigating the association with biological factors.

##### Risk factors

In nursing home patients with cognitive impairment, BPS were associated with the psychosocial environment, in addition to dementia type and stage and medication use [24]. In dementia patients, psychosis has been associated with age, illness duration and functional impairment, whereas results are weak or inconsistent for sociodemographic variables [27].

In two moderate quality reviews of the older population by Huang *et al.*, depression was reported to be common in those with poor self-rated health, disability and chronic disease, including stroke, sensory impairment, cardiac disease or chronic lung disease [43,44]. Depression is more common in women, and has been associated with many risk factors, including other diseases, low social support, cognitive impairment, disability, prior depression and bereavement [20,29-31,49,50]. Vink *et al. *report that health factors were less clearly related to anxiety than to depression [49]. Psychosocial associations of other BPS have not been reviewed.

More research is recommended on risk factors for depression and randomised controlled trials to investigate if manipulation of risk factors reduces the onset of BPS.

#### Outcomes and care

Some evidence suggests that BPS predicts nursing home placement in those with dementia [51]. Burden of care, caregiver's general health and caregiver depression scores have been associated with BPS, but perhaps caregiver's perception of the BPS and caregiver's social and psychological resources prior to institutionalisation are more important factors [52]. Lee *et al. *concluded that there was no consensus regarding the association between BPS and increased mortality in individuals with dementia [54]. Studies of associations between delusions in dementia and functional outcome had inconsistent results [55]. Finally, depression has been associated with decreased health related quality of life in dementia, although the size of the association was moderate [53].

Depression in the general older population has been associated with increased health care utilisation and expenditure [30]. In addition, anxiety in the elderly has been reported to have negative consequences independently of depression [32].

The reviews recommend more research with better measurements of determinants and outcomes and more sophisticated techniques to analyse the association with disease outcomes.

### Summary: limitations and recommendations

**Figure 2:**
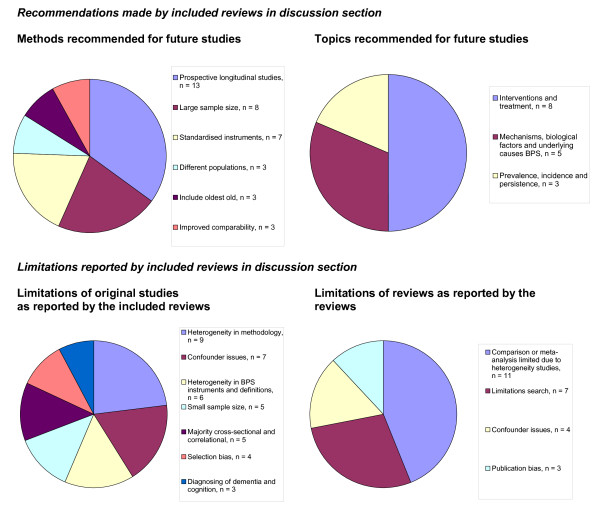
**Overview of the recommendations and limitations reported by the included reviews**. In total, 36 reviews were included in the review of reviews. Only recommendations and limitations reported by three or more reviews are included in the figures. Reviews that make multiple recommendations or limitations may be included more than once.

Overall, the reviews reported several limitations of the original studies, including heterogeneity in methodology, insufficient adjustment for confounders, heterogeneity in BPS instruments and definitions, small sample size and that most studies were cross-sectional (Figure 2). This led to recommendations for prospective longitudinal studies, with a large sample size and using standardised BPS instruments and definitions. The following topics for future research were most often recommended, including intervention and treatment, mechanisms and underlying causes of BPS and the prevalence, incidence and persistence of BPS. Many reviews reported that comparison of results was limited due to heterogeneity of the included studies.

### Gaps

**Figure 3:**
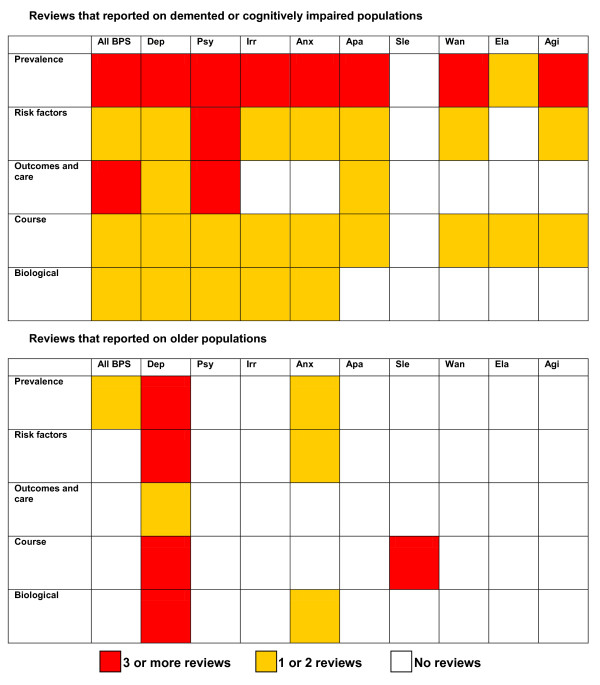
**Number of reviews that reported on each symptom by the topic of the review**. Agg, aggression; Agi, agitation; Anx, anxiety; BPS, behavioural and psychological symptoms; Dep, depressive symptoms; Ela, elation; Psy, psychosis; Sle, sleep problems; Wan, wandering; In total 36 reviews were included in the review of reviews. Reviews that report on multiple topics or more than one BPS are included more than once.

Figure 3 gives an overview of the number of reviews that studied each BPS by the topic of the review. Most of the included reviews focused on individuals with dementia or cognitive impairment studied depression or psychotic symptoms, and they most often studied the prevalence of symptoms. Of the reviews of older populations, almost all studied depressive symptoms and some studied sleep problems or anxiety, whereas the other BPS were not investigated.

## Discussion

### Summary of findings

• **Prevalence**. BPS occur in non-demented, cognitively impaired and demented populations, but there are large variations in reported prevalence estimates.

• **Biological associations**. Biological factors associated with BPS include health status, homocysteine levels and vascular disease.

• **Risk factors**. Associations have been found with psychosocial environment and dementia severity. Results are inconsistent, especially for socio-demographic factors, and differ between symptoms.

• **Outcomes and care**. BPS have been associated with increased burden of care, decreased caregiver's general health and increased caregiver depression scores, but these conclusions come from a limited number of studies. Findings in relation to BPS and institutionalisation, quality of life and disease outcome are generally inconsistent.

### Limitations of reviews

Over 97,000 articles have been published on BPS in recent years (up to July 2011). However, in total, only 36 systematic reviews were identified, published from 1989 to 2011, covering 6 to 244 papers, with only 10 reviews, including a meta-analysis. These covered a wide area of research and included studies with large differences in population characteristics, recruitment and definition of BPS. Depressive symptoms were most widely reviewed. Other symptoms, such as apathy, irritability, wandering and elation, are typically ignored, especially in studies of the older population. Aggression, psychosis and wandering have been identified as the BPS that are most difficult to cope with by caregivers [61]. Not all areas of BPS research have yet been adequately reviewed. For example, no review has yet appeared on the neuropathology underpinning BPS, although there are several recent studies on this subject and many possible biological mechanisms may underlie BPS, such as Alzheimer's disease pathology, lower neuronal counts, neurotransmitter changes, genetic risk factors and abnormal neuroendocrinology [62-64]. A high quality systematic review of the biological underpinnings of BPS would give direction to further research, particularly with regard to treatment development.

The quality of the included reviews as measured with the AMSTAR tool was generally low. All except one did not report if the research question and inclusion criteria were established before the conduct of the review, or they did not provide a research question and/or inclusion criteria. Many reviews did not have two independent researchers who selected the studies and extracted the data, or did not report this. None of the reviews reported the potential conflict of interest of the included studies. Furthermore, the inclusion of grey literature and if the search was supplemented by consulting current contents, reviews, textbooks, specialised registers or experts were rarely reported.

Better and more systematic reporting would make it easier to compare the characteristics, results and strengths and limitations of reviews, and the use of reporting checklists, such as PRISMA, is recommended [65]. More details on the populations covered by the review and the characteristics of included studies would be particularly helpful.

Recommendations for future research made by the reviews were variable, with many recommendations made only by one or two reviews. Only more general recommendations were made by several reviews. This has been previously reported by Clark *et al.*, who examined 2,535 Cochrane reviews and found that the characterisation of the needs for future research was less than explicit [66]. In addition, it has been previously found that reviews tend to be too optimistic when drawing conclusions from their results [67,68]. It has been recommended that research gaps should be identified more systematically, rating the reasons of research gaps in terms of population, intervention, comparison, outcome and setting (PICOS), including insufficient information, biased information, inconsistency or not the right information [69], although this tool has been designed for reviews of intervention studies and may not be suitable for reviews of observational studies.

### Limitations of original studies

The reviews have included individual research studies with a large degree of heterogeneity in study design (for example, definition and measurement of BSP, level of cognitive impairment, population characteristics and recruitment of participants), making cross study comparisons difficult. Many different instruments are used to measure BPS, including the Neuropsychiatric Inventory (NPI) [70], CERAD-BRSD [71] and the Behavioral Pathology in Alzheimer's Disease scale (BEHAVE-AD) [72]. Symptom-specific instruments are also used, including the Geriatric Depression Scale (GDS) for depressive symptoms [73]. Some instruments use self-ratings (for example, GDS), while others are based on a caregiver interview (for example, NPI). Across instruments there is no consistency in the definition and severity of the symptoms.

Some studies focused on the general older population, while others selected only those with dementia or MCI. Even within impaired groups large differences exist in the measurement of cognitive function. Some study populations were divided by cognitive status (for example, normal, mild or other cognitive impairment and dementia) using diagnostic criteria applied independently of BPS. It may be that the phenotypes of BPS (for example, the causes of depression or agitation) have a different basis at different stages of cognitive impairment and, therefore, studies using different groups will have discrepant findings. Other features, such as the age and gender of participants, and the setting where they were recruited, also vary. If samples are not representative of the population (either that of older people in the community or people with dementia) conclusions may be difficult to generalise.

### Limitations of our review of review

There is no Mesh or Emtree search term for BPS, so search terms for individual symptoms and text searches had to be combined. As many different terms are used to describe BPS, it is possible that we may have missed relevant reviews, though we have checked all reference lists of relevant papers. One author undertook data selection and extraction.

Many different definitions of BPS have been used in the literature. Here we used symptom definitions as used by the most commonly used instruments to measure BPS, including the NPI. However, symptom definitions may overlap and there was heterogeneity in symptom definitions that were used by the reviews. Depression is especially problematic. All of the reviews included here that studied depression included studies investigating major depression as well as studies investigating depressive symptoms. In the original studies, decisions about depression can be made at recruitment, when applying the inclusion and exclusion criteria or during measurement at baseline and follow-up. In some studies, only people with or without depressive symptoms or major depression are recruited, or participants with major depression can be excluded from the study at the participant selection stage. At the data collection stage, the definition of depression can include both major depression and minor depression using a cut-off score (for example, a CES-D score higher than 16); it can include both depressive symptoms and major depression separately (for example, DSM definitions for major and minor depression or CES-D scores 16 to 20 and CES-D higher than 20), or either depressive symptoms only (for example, using the Neuropsychiatric Inventory to measure depressive symptoms in the context of BPSD) or major depression only (for example, DSM definition for major depression).

By reviewing reviews and not the original studies, it is likely that noise has been introduced. Furthermore, with the diverse and generally qualitative outcomes of the majority of reviews, we were unable to combine results quantitatively and, therefore, a meta-meta-analysis was not possible.

### Recommendations for future research

Recommendations for future reviews on BPS:

• More reviews on BPS are needed, for example, on neuropathological associations

• Improve the quality of reporting of reviews

• Choose reviews that include all BPS not just symptoms of depression/psychosis

• Clearer reporting, using a checklist tool, for example, PRISMA

• Clearer and more specific recommendations for future research, for example, PICOS if applicable

Recommendations for original research on BPS:

• Prospective longitudinal studies

• Large sample size

• Standardised instruments for BPS

• Wide age range, including oldest old

• Improved comparability of results; report study characteristics clearly

## Conclusions

No clear conclusions could be made on the prevalence, biology, risk factors and outcomes of BPS. However, by pulling together this wide variety of research, we have been able to give an overview of the recommendations, limitations and gaps of current research in BPS that may inform future research. More high quality reviews including all BPS, not just depressive symptoms, are needed. Future original research should include longitudinal studies with larger sample sizes to further assess the complex relations between BPS, cognitive function and psychological, social and biological factors. One of the main questions raised by the reviews is how best to define and measure BPS within and across populations (that is, different levels of cognitive function, population vs. clinical based). A wider use of the most frequently cited instruments to measure BPS, such as the NPI, would improve comparability. Studies should report clearly the characteristics of their population, the inclusion and exclusion criteria that were used and how they defined BPS, particularly depression. A better understanding of BPS, including their definition, evaluation, underlying mechanisms, risk factors, prevalence and progression will have important implications for prevention and treatment. This has the potential to significantly improve quality of life in people with dementia and their carers as well has having other positive impacts, for example, on economics, institutionalisation and worsening of both cognitive and non-cognitive symptoms.

## Abbreviations

AMSTAR: a measurement tool for the 'assessment of multiple systematic reviews'; APOE E: Apolipoprotein E; BEHAVE-AD: Behavioral Pathology in Alzheimer's Disease scale; BPS: behavioural and psychological Symptoms; BPSD: behavioural and psychological symptoms of dementia; BSRS: Brief Behaviour Symptom Rating Scale; CERAD-BRSD: Behavior Rating Scale for Dementia of the Consortium to Establish a Registry for Alzheimer's Disease; CES-D: Centre for Epidemiological Studies Depression scale; COMT: Catechol-O-methyltransferase; DSM: Diagnostic and Statistical Manual of Mental Disorders; GDS: Geriatric Depression Scale; MAO-A: Mono-amine oxidase A; MCI: Mild cognitive impairment; NPI: Neuropsychiatric Inventory; PICOS: population, intervention, comparison, outcome and setting; PRISMA: Cochrane Collaboration and the Preferred Reporting Items for Systematic Reviews and Meta-Analyses.

## Competing interests

The authors declare that they have no competing interests.

## Authors' contributions

RvdL participated in the conception and design of the study, performed the literature search, interpreted and summarised the data, and drafted the manuscript. BS and GS participated in interpretation of the data and critically revised the manuscript. TD and CB participated in the conception and design of the study, interpretation and visualisation of the results and critically revised the manuscript. All authors read and approved the final manuscript.

## Supplementary Material

Additional file 1 **Search terms (Embase and Medline, 29 March 2012)**. An overview of the search terms that were used. Click here for file

## References

[B1] Savva GM Zaccai J Matthews FE Davidson JE McKeith I Brayne C Prevalence, correlates and course of behavioural and psychological symptoms of dementia in the population Br J Psychiatry 2009 194 212 219 10.1192/bjp.bp.108.049619 19252147

[B2] National Institute for Health and Clinical Excellence Dementia - Supporting people with Dementia and their Carers in Health and Social Care 2006

[B3] Department of Health (UK) Living Well with Dementia: A National Dementia Strategy 2009

[B4] Smith V Devane D Begley CM Clarke M Methodology in conducting a systematic review of systematic reviews of healthcare interventions BMC Med Res Methodol 2011 11 15 10.1186/1471-2288-11-15 21291558 PMC3039637

[B5] Kraepelin E Psychiatrie: ein Lehrbuch für studierende und Ärtze 1896 Leipzig: J.A. Barth

[B6] Roth M The natural history of mental disorder in old age J Ment Sci 1955 101 281 301 13243044 10.1192/bjp.101.423.281

[B7] Finkel SI Costa e Silva J Cohen G Miller S Sartorius N Behavioral and psychological signs and symptoms of dementia: a consensus statement on current knowledge and implications for research and treatment Int Psychogeriatr 1996 8 Suppl 3 497 500 9154615 10.1017/s1041610297003943

[B8] Alzheimer A Über einen eigenartigen schweren Er Krankungsprozeb der Himrinde Neurol Zbl 1906 23 1129 1136

[B9] Reisberg B Borenstein J Salob SP Ferris SH Franssen E Georgotas A Behavioral symptoms in Alzheimer's disease: phenomenology and treatment J Clin Psychiatry 1987 48 Suppl 9 15 3553166

[B10] Moher D Liberati A Tetzlaff J Altman DG Preferred reporting items for systematic reviews and meta-analyses: the PRISMA Statement Open Med 2009 3 e123 e130 10.2174/1874306400903010123 21603045 PMC3090117

[B11] Shea BJ Grimshaw JM Wells GA Boers M Andersson N Hamel C Porter AC Tugwell P Moher D Bouter LM Development of AMSTAR: a measurement tool to assess the methodological quality of systematic reviews BMC Med Res Methodol 2007 15 7 10 10.1186/1471-2288-7-10 PMC1810543 17302989

[B12] Shea BJ Hamel C Wells GA Bouter LM Kristjansson E Grimshaw J Henry DA Boers M AMSTAR is a reliable and valid measurement tool to assess the methodological quality of systematic reviews J Clin Epidemiol 2009 62 1013 1020 10.1016/j.jclinepi.2008.10.009 19230606

[B13] Cole MG Bellavance F The prognosis of depression in old age Am J Geriatr Psychiatry 1997 5 4 14 9169240

[B14] Christensen H Griffiths K Mackinnon A Jacomb P A quantitative review of cognitive deficits in depression and Alzheimer-type dementia J Int Neuropsychol Soc 1997 3 631 651 9448376

[B15] Cole MG Bellavance F Depression in elderly medical inpatients: a meta-analysis of outcomes Can Med Assoc J 1997 157 1055 1060 9347776 PMC1228261

[B16] Cole MG Does depression in older medical inpatients predict mortality? A systematic review Gen Hosp Psychiatry 2007 29 425 430 10.1016/j.genhosppsych.2007.07.002 17888809

[B17] Henry JD Crawford JR A meta-analytic review of verbal fluency deficits in depression J Clin Exp Neuropsychol 2005 27 78 101 10.1080/138033990513654 15814444

[B18] Nakajima GA Wenger NS Quality indicators for the care of depression in vulnerable elders J Am Geriatr Soc 2007 55 S302 S311 17910551 10.1111/j.1532-5415.2007.01336.x

[B19] Savoie I Morettin D Green CJ Kazanjian A Systematic review of the role of gender as a health determinant of hospitalization for depression Int J Technol Assess Health Care 2004 20 115 127 15209172 10.1017/s026646230400090x

[B20] Chen R Copeland JR Wei L A meta-analysis of epidemiological studies in depression of older people in The People's Republic of China Int J Geriatr Psychiatry 1999 14 821 830 10.1002/(SICI)1099-1166(199910)14:10<821::AID-GPS21>3.0.CO;2-0 10521881

[B21] Monastero R Mangialasche F Camarda C Ercolani S Camarda R A systematic review of neuropsychiatric symptoms in mild cognitive impairment J Alzheimer's Dis 2009 18 11 30 19542627 10.3233/JAD-2009-1120

[B22] Apostolova LG Cummings JL Neuropsychiatric manifestations in mild cognitive impairment: A systematic review of the literature Dementia Geriatr Cogn Disord 2008 25 115 126 10.1159/000112509 18087152

[B23] Seitz D Purandare N Conn D Prevalence of psychiatric disorders among older adults in long-term care homes: a systematic review Int Psychogeriatr 2010 22 1025 1039 10.1017/S1041610210000608 20522279

[B24] Zuidema S Koopmans R Verhey F Prevalence and predictors of neuropsychiatric symptoms in cognitively impaired nursing home patients J Geriatr Psychiatry Neurol 2007 20 41 49 10.1177/0891988706292762 17341770

[B25] Shub D Ball V Abbas AAA Gottumukkala A Kunik ME The link between psychosis and aggression in persons with dementia: a systematic review Psychiatr Q 2010 81 97 110 10.1007/s11126-009-9121-7 20058077

[B26] Wragg RE Jeste DV Overview of depression and psychosis in Alzheimer's disease Am J Psychiatry 1989 146 577 587 2653053 10.1176/ajp.146.5.577

[B27] Ropacki SA Jeste DV Epidemiology of and risk factors for psychosis of Alzheimer's disease: a review of 55 studies published from 1990 to 2003 Am J Psychiatry 2005 162 2022 2030 10.1176/appi.ajp.162.11.2022 16263838

[B28] Luppa M Sikorski C Luck T Ehreke L Konnopka A Wiese B Weyerer S Konig HH Riedel-Heller SG Age- and gender-specific prevalence of depression in latest-life - Systematic review and meta-analysis J Affective Disord 2012 136 212 221 10.1016/j.jad.2010.11.033 21194754

[B29] Beekman AT Copeland JR Prince MJ Review of community prevalence of depression in later life Br J Psychiatry 1999 174 307 311 10.1192/bjp.174.4.307 10533549

[B30] Meeks TW Vahia IV Lavretsky H Kulkarni G Jeste DV A tune in "a minor" can "b major": a review of epidemiology, illness course, and public health implications of subthreshold depression in older adults J Affective Disord 2011 129 126 142 10.1016/j.jad.2010.09.015 PMC3036776 20926139

[B31] Djernes JK Prevalence and predictors of depression in populations of elderly: a review Acta Psychiatr Scand 2006 113 372 387 10.1111/j.1600-0447.2006.00770.x 16603029

[B32] Alwahhabi F Anxiety symptoms and generalized anxiety disorder in the elderly: a review Harv Rev Psychiatry 2003 11 180 193 10.1080/10673220303944 12944126

[B33] Jorm AF van Duijn CM Chandra V Fratiglioni L Graves AB Heyman A Kokmen E Kondo K Mortimer JA Rocca WA Psychiatric history and related exposures as risk factors for Alzheimer's disease: a collaborative re-analysis of case-control studies Int J Epidemiol 1991 20 Suppl 2 S43 S47 1917269 10.1093/ije/20.supplement_2.s43

[B34] Verkaik R Nuyen J Schellevis F Francke A The relationship between severity of Alzheimer's disease and prevalence of comorbid depressive symtoms and depression: a systematic review Int J Geriatr Psychiatry 2007 22 1063 1086 10.1002/gps.1809 17457960

[B35] Huang CQ Wang ZR Li YH Xie YZ Liu QX Cognitive function and risk for depression in old age: a meta-analysis of published literature Int Psychogeriatr 2011 23 516 525 10.1017/S1041610210000049 20937170

[B36] Jorm AF History of depression as a risk factor for dementia: an updated review Aust N Z J Psychiatry 2001 35 776 781 10.1046/j.1440-1614.2001.00967.x 11990888

[B37] Jorm AF Is depression a risk factor for dementia or cognitive decline? A review Gerontology 2000 46 219 227 10.1159/000022163 10859462

[B38] Ohayon MM Carskadon MA Guilleminault C Vitiello MV Meta-analysis of quantitative sleep parameters from childhood to old age in healthy individuals: developing normative sleep values across the human lifespan Sleep 2004 27 1255 1273 15586779 10.1093/sleep/27.7.1255

[B39] Floyd JA Janisse JJ Jenuwine ES Ager JW Changes in REM-sleep percentage over the adult lifespan Sleep 2007 30 829 836 17682652 10.1093/sleep/30.7.829 PMC1978369

[B40] Floyd JA Medler SM Ager JW Janisse JJ Age-related changes in initiation and maintenance of sleep: a meta-analysis Res Nurs Health 2000 23 106 117 10.1002/(SICI)1098-240X(200004)23:2<106::AID-NUR3>3.0.CO;2-A 10782869

[B41] Kuo HK Sorond FA Chen JH Hashmi A Milberg WP Lipsitz LA The role of homocysteine in multisystem age-related problems: A systematic review J Gerontol Ser A Biol Sci Med Sci 2005 60 1190 1201 10.1093/gerona/60.9.1190 16183962

[B42] Flirski M Sobow T Kloszewska I Behavioural genetics of Alzheimer's disease: A comprehensive review Arch Med Sci 2011 7 195 210 22291757 10.5114/aoms.2011.22068 PMC3258720

[B43] Huang CQ Dong BR Lu ZC Yue JR Liu QX Chronic diseases and risk for depression in old age: a meta-analysis of published literature Ageing Res Rev 2010 9 131 141 10.1016/j.arr.2009.05.005 19524072

[B44] Chang-Quan H Xue-Mei Z Bi-Rong D Zhen-Chan L Ji-Rong Y Qing-Xiu L Health status and risk for depression among the elderly: a meta-analysis of published literature Age Ageing 2010 39 23 30 10.1093/ageing/afp187 19903775

[B45] Almeida OP McCaul K Hankey GJ Norman P Jamrozik K Flicker L Homocysteine and depression in later life Arch Gen Psychiatry 2008 65 1286 1294 10.1001/archpsyc.65.11.1286 18981340

[B46] Stetler C Miller GE Depression and hypothalamic-pituitary-adrenal activation: A quantitative summary of four decades of research Psychosom Med 2011 73 114 126 10.1097/PSY.0b013e31820ad12b 21257974

[B47] Kuo HK Yen CJ Chang CH Kuo CK Chen JH Sorond F Relation of C-reactive protein to stroke, cognitive disorders, and depression in the general population: Systematic review and meta-analysis Lancet Neurol 2005 4 371 380 10.1016/S1474-4422(05)70099-5 15907742

[B48] Camus V Kraehenbuhl H Preisig M Bula CJ Waeber G Geriatric depression and vascular diseases: what are the links? J Affective Disord 2004 81 1 16 10.1016/j.jad.2003.08.003 15183594

[B49] Vink D Aartsen MJ Schoevers RA Risk factors for anxiety and depression in the elderly: a review J Affective Disord 2008 106 29 44 10.1016/j.jad.2007.06.005 17707515

[B50] Cole MG Dendukuri N Risk factors for depression among elderly community subjects: a systematic review and meta-analysis Am J Psychiatry 2003 160 1147 1156 10.1176/appi.ajp.160.6.1147 12777274

[B51] Gaugler JE Yu F Krichbaum K Wyman JF Predictors of nursing home admission for persons with dementia Med Care 2009 47 191 198 10.1097/MLR.0b013e31818457ce 19169120

[B52] Black W Almeida OP A systematic review of the association between the Behavioral and Psychological Symptoms of Dementia and burden of care Int Psychogeriatr 2004 16 295 315 10.1017/S1041610204000468 15559754

[B53] Banerjee S Samsi K Petrie CD Alvir J Treglia M Schwam EM de Valle M What do we know about quality of life in dementia? A review of the emerging evidence on the predictive and explanatory value of disease specific measures of health related quality of life in people with dementia Int J Geriatr Psychiatry 2009 24 15 24 10.1002/gps.2090 18727132

[B54] Lee M Chodosh J Dementia and life expectancy: what do we know? J Am Med Dir Assoc 2009 10 466 471 10.1016/j.jamda.2009.03.014 19716062

[B55] Fischer CE Verhoeff NP Churchill K Schweizer TA Functional outcome in delusional Alzheimer disease patients: a systematic review Dement Geriatr Cogn Disord 2009 27 105 110 10.1159/000194659 19169029

[B56] Luppa M Sikorski C Luck T Ehreke L Konnopka A Wiese B Weyerer S Konig HH Riedel-Heller SG Age- and gender-specific prevalence of depression in latest-life--systematic review and meta-analysis J Affect Disord 2012 136 212 221 10.1016/j.jad.2010.11.033 21194754

[B57] Chang-Quan H Xue-Mei Z Bi-Rong D Zhen-Chan L Ji-Rong Y Qing-Xiu L Health status and risk for depression among the elderly: a meta-analysis of published literature Age Ageing 2010 39 23 30 10.1093/ageing/afp187 19903775

[B58] Beekman AT Copeland JR Prince MJ Review of community prevalence of depression in later life Br J Psychiatry 1999 174 307 311 10.1192/bjp.174.4.307 10533549

[B59] Ropacki SA Jeste DV Epidemiology of and risk factors for psychosis of Alzheimer's disease: a review of 55 studies published from 1990 to 2003 Am J Psychiatry 2005 162 2022 2030 10.1176/appi.ajp.162.11.2022 16263838

[B60] Ownby RL Crocco E Acevedo A John V Loewenstein D Depression and risk for Alzheimer disease: systematic review, meta-analysis, and metaregression analysis Arch Gen Psychiatry 2006 63 530 538 10.1001/archpsyc.63.5.530 16651510 PMC3530614

[B61] International Psychogeriatric Association (IPA) Behavioral and Psychological Symptoms of Dementia (BPSD) Educational Pack 2002

[B62] Serra L Perri R Cercignani M Spano B Fadda L Marra C Carlesimo GA Caltagirone C Bozzali M Are the behavioral symptoms of Alzheimer's disease directly associated with neurodegeneration? J Alzheimers Dis 2010 21 627 639 20555138 10.3233/JAD-2010-100048

[B63] Berlow YA Wells WM Ellison JM Sung YH Renshaw PF Harper DG Neuropsychiatric correlates of white matter hyperintensities in Alzheimer's disease Int J Geriatr Psychiatry 2010 25 780 788 19946864 10.1002/gps.2418 PMC3975914

[B64] Staekenborg SS Gillissen F Romkes R Pijnenburg YA Barkhof F Scheltens P van der Flier WM Behavioural and psychological symptoms are not related to white matter hyperintensities and medial temporal lobe atrophy in Alzheimer's disease Int J Geriatr Psychiatry 2008 23 387 392 10.1002/gps.1891 17907266

[B65] Moher D Liberati A Tetzlaff J Altman DG Preferred reporting items for systematic reviews and meta-analyses: the PRISMA Statement Open Med 2009 3 e123 e130 10.2174/1874306400903010123 21603045 PMC3090117

[B66] Clarke L Clarke M Clarke T How useful are Cochrane reviews in identifying research needs? J Health Serv Res Policy 2007 12 101 103 10.1258/135581907780279648 17407660

[B67] Olsen O Middleton P Ezzo J Gotzsche PC Hadhazy V Herxheimer A Kleijnen J McIntosh H Quality of Cochrane reviews: assessment of sample from 1998 BMJ 2001 323 829 832 10.1136/bmj.323.7317.829 11597965 PMC57800

[B68] Schunemann H Oxman A Vist G Higgins J Deeks J Glasziou P Guyatt G Higgins J, Green S Chapter 12: Interpreting results and drawing conclusions Cochrane Handbook for Systematic Reviews of Interventions (Version 5.1.0) 2011 Oxford: The Cochrane Collaboration 359 388

[B69] Robinson KA Saldanha IJ McKoy NA Development of a framework to identify research gaps from systematic reviews J Clin Epidemiol 2011 64 1325 1330 10.1016/j.jclinepi.2011.06.009 21937195

[B70] Cummings JL Mega M Gray K Rosenberg-Thompson S Carusi DA Gornbein J The Neuropsychiatric Inventory: comprehensive assessment of psychopathology in dementia Neurology 1994 44 2308 2314 10.1212/WNL.44.12.2308 7991117

[B71] Tariot PN CERAD behavior rating scale for dementia Int Psychogeriatr 1996 8 Suppl 3 317 320 9154581 10.1017/s1041610297003542

[B72] Reisberg B Auer SR Monteiro IM Behavioral pathology in Alzheimer's disease (BEHAVE-AD) rating scale Int Psychogeriatr 1996 8 Suppl 3 301 308 9154579 10.1097/00019442-199911001-00147

[B73] Yesavage JA Geriatric Depression Scale Psychopharmacol Bull 1988 24 709 711 3249773

