# Supplementary material for: Systematic reviews on behavioural and psychological symptoms in the older or demented population
Source: Alzheimers Res Ther. 2012 Jul 11;4(4):28. doi: 10.1186/alzrt131 (PMC3506942; doi:10.1186/alzrt131)
Supplement: Additional file 1 — Search terms (Embase and Medline, 29 March 2012). An overview of the search terms that were used. [file alzrt131-S1.DOCX]

**Additional file A Search terms (Embase and Medline, 29 March 2012)**

**BPSD**

**Text[ti ab]**

“neuropsychiatric symptoms”

“neuro-psychiatric symptoms”

“psycho-behavioral symptoms”

“psycho-behavioural

Symptoms”

“psychiatric symptoms”

“Behavioral symptoms”

“behavioural symptoms” “psychological symptoms” “disruptive behaviour” “disruptive behaviour”

“non-cognitive symptoms”

“neuropsychological symptoms”

“bpsd”

**Total (OR):**

18,254

Aged: 3,539

Dem: 3,808

Total: 5,622

Aged: 17

Dem: 136

Total: 137

Aged: 17

Dem: 123

Total: 124

Aged: 6

Dem: 28

Total: 29

**AGITATI**

**Emtree**

restlessness

**Text[tiab]**

agitation

agitated

**Total (OR):**

20,990

Aged: 3,330

Dem: 2,390

Total: 4,404

Aged: 34

Dem: 100

Total: 117

Aged: 33

Dem: 99

Total: 115

Aged: 3

Dem: 14

Total: 15

**ELATION**

**Emtree**

Euphoria

**Text[tiab]**

euphoria

elation disinhibition

laughter

**Total (OR):**

10,393

Aged: 890

Dem: 743

Total: 1,324

Aged: 3

Dem: 11

Total: 14

Aged: 2

Dem: 11

Total: 13

Aged: 0

Dem: 3

Total: 3

**WANDER**

**Emtree**

Wandering behaviour

**Text[tiab]**

wandering

“stalking”

“getting lost”

“Aberrant motor behaviour”

**Total (OR):** 3,040

Aged: 559

Dem: 523

Total: 747

Aged: 3

Dem: 15

Total: 15

Aged: 3

Dem: 15

Total: 15

Aged: 0

Dem: 3

Total: 3

**DEPRES**

**Emtree**

Depression

**Text[ti ab]**

dysphoria

depress*

**Total (OR):** 451,604

Aged: 63,156

Dem: 17,289

Total: 73,365

Aged: 311

Dem: 364

Total: 627

Aged: 298

Dem: 346

Total: 597

Aged: 134

Dem: 156

Total: 266

**PSYCHO**

**Emtree**

Psychotic disorders

Delusions

Paranoid behaviour

Hallucinations

**Text[tiab]**

psychosis

psychotic

delusion*

hallucinat*

misidentification

**Total (OR):**

82,085

Aged: 9,936

Dem: 6,018

Total: 13,489

Aged: 53

Dem: 145

Total: 179

Aged: 50

Dem: 133

Total: 164

Aged: 15

Dem: 33

Total: 42

**IRRITABI**

**Emtree**

Irritable mood

Aggression

Anger

**Text[tiab]**

irritability

lability

mood change*

aggression

rage

“catastrophic reactions”

anger

angry

complaining

negativism

screaming

**Total (OR):**

102,393

Aged: 9,956

Dem: 4,350

Total: 12,231

Aged: 53

Dem: 129

Total: 156

Aged: 50

Dem: 126

Total: 150

Aged: 10

Dem: 24

Total: 30

**SLEEP**

**Emtree**

Sleep disorder

Sleep

Sleep Apnea syndrome

**Text[tiab]**

Sleep

**Total (OR):**

208,981

Aged: 26,260

Dem: 6,106

Total: 30,592

Aged: 158

Dem: 222

Total: 360

Aged: 157

Dem: 211

Total: 348

Aged: 42

Dem: 45

Total: 82

**APATHY**

**Emtree**

**Text[tiab]**

Apathy

“Lack of interest”

**Total (OR):**

4,287

Aged: 1,030

Dem: 1,114

Total: 1,618

Aged: 4

Dem: 23

Total: 24

Aged: 4

Dem: 22

Total: 23

Aged: 4

Dem: 7

Total: 8

**ANXIETY**

**Emtree**

Anxiety disorder

Anxiety

**Text[tiab**

anxiety

anxious

**Total (OR):**

236,496

Aged: 27,044

Dem: 5,742

Total: 29,564

Aged: 125

Dem: 163

Total: 272

Aged: 121

Dem: 159

Total: 264

Aged: 42

Dem: 74

Total: 110

**Dementia or older population:** AND Aged OR Dementia

**Systematic review** Cochrane review, systematic review or meta-analysis

**Language = english**

**Not pharmacological treatment**

NOT clinical trial NOT randomized controlled trial NOT pharmaco*:ti :ab NOT treat*:ti NOT therap*:ti NOT drug*:ti :ab NOT antipsychot*:ti :ab NOT antidepress*:ti :ab NOT anxiolytic:ti :ab NOT  anticonvuls*:ti :ab NOT  cholinesterase:ti :ab NOT  memantine:ti :ab NOT  neuropharmaco*:ti NOT  psychopharmaco*:ti NOT haloperidol:ti :ab NOT  thiothixene:ti :ab NOT risperidone:ti :ab NOT clozapine:ti :ab NOT  olanzapine:ti :ab NOT quetiapine:ti :ab NOT neuroleptic*:ti :ab NOT  trazodone:ti :ab NOT  'selective serotonin reuptake inhibitors':ti :ab NOT  ssri:ti :ab NOT  moclobemide:ti :ab NOT  benzodiazepine*:ti :ab NOT  anticonvulsant:ab :ti NOT  'valproic acid':ti :ab NOT  carbamazepine:ti :ab
